# Supplementary material for: Engineered nanointerfaces for microfluidic isolation and molecular profiling of tumor-specific extracellular vesicles
Source: Nat Commun. 2018 Jan 12;9:175. doi: 10.1038/s41467-017-02261-1 (PMC5766611; doi:10.1038/s41467-017-02261-1)
Supplement: Supplementary file 1 — Supplementary Information [file 41467_2017_2261_MOESM1_ESM.pdf]

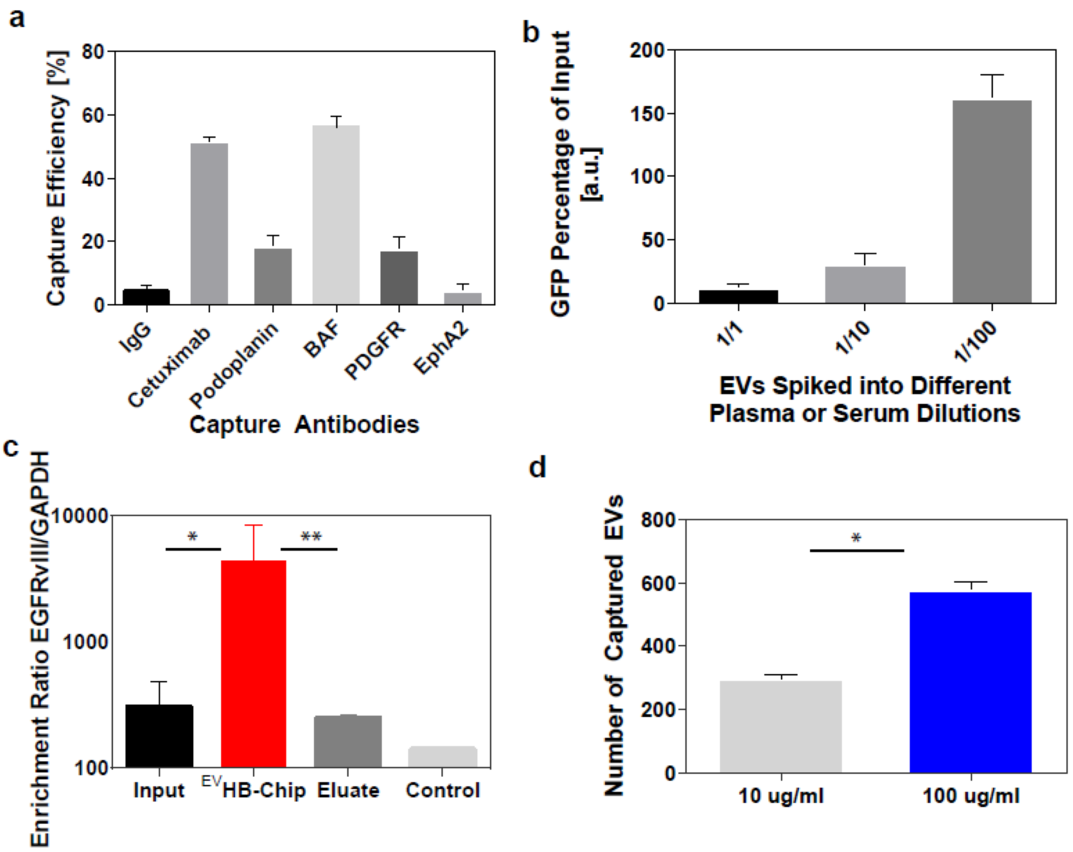

Supplementary Figure 1. (a) Different antibodies were selected to test their specificity towards GBM cell lines to identify antibody candidates for the specific capture of tumor EVs. GBM20/3 cells were spiked at 1000 cells/mL in PBS. **(b)** EVs from GBM20/3 palmGFP cells were spiked into plasma to quantify tumor-specific message at different dilution ratios. **(c)** Comparison of the obtained enrichment ratios at the various stages of sample processing in the ^{EV}HB-Chip (n = 3 technical replicates; ± s.e.m., *, ** $p < 0.05$, 1-way ANOVA). **(d)** Effect of antibody concentration on the capture of tumor-specific EVs (n = 3 technical replicates; ± s.e.m., * $p < 0.05$, student t-test).

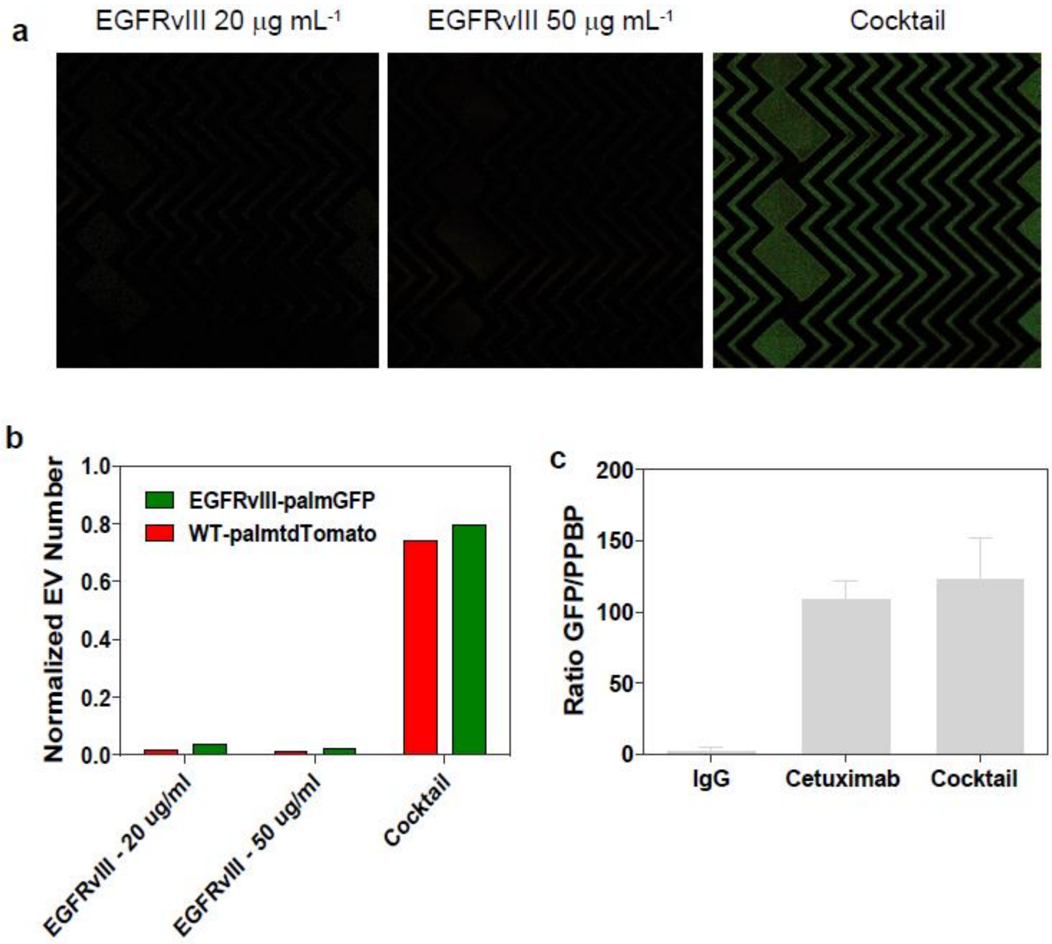

Supplementary Figure 2. **(a)** Comparative confocal microscopy images of tumor EVs captured on the surface of the ^{EV}HB-Chip with different isolation antibodies (e.g., concentration and different antibodies). **(b)** Normalized quantification of captured EVs on the surface of the ^{EV}HB-Chip. **(c)** Comparison of the tumor-specific EV capture for different antibodies on the surface of the ^{EV}HB-Chip. For data shown in (a) and (b) EVs were obtained from Gli36 cells; data in (c) was obtained using EVs from GBM20/3 with palmGFP. The cocktail of antibodies used for these experiments consisted of EGFRvIII, BAF231, PDGFR, Podoplanin, and Cetuximab; each antibody at a concentration of 10ug/mL. Cetuximab alone was used at a concentration of 20 μg mL^{-1}.

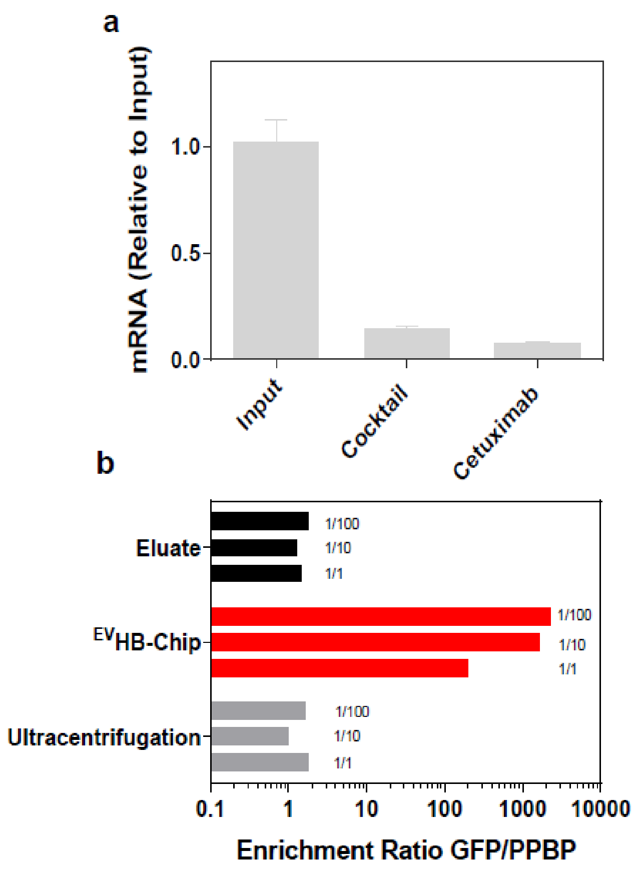

Supplementary Figure 3. (a) Comparison of retention of non-specific EVs on the ^{EV}HB-Chip by RT-PCR quantification of the pro-platelet basic protein (PPBP). The total amount of PBPP present in EVs isolated using ultracentrifugation of bulk plasma ('input') is compared to the signal present in EVs isolated on the device. The same cocktail of antibodies used in **Supplementary Figure 2** was used for these experiments. Cetuximab was used at a concentration of 20 μg/mL. **(b)** Comparison of enrichment ratios for different dilutions of plasma (1/1, 1/10, 1/100) using an ultracentrifugation method and the ^{EV}HB-Chip. GBM20/3-GFP EVs spiked into plasma were used and Cetuximab was used as the capture antibody at a concentration of 20 μg mL^{-1}.

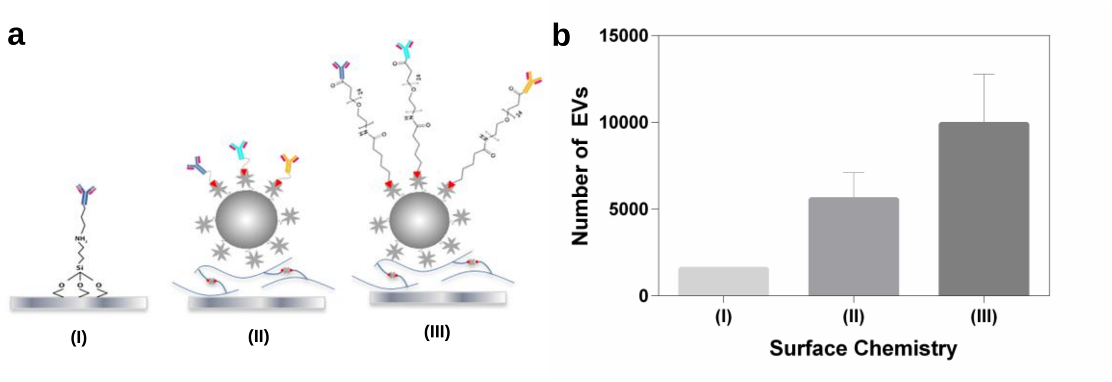

Supplementary Figure 4. (a) Schematic representation of the different chemistries used for capture antibody immobilization on the surface of the ^{EV}HB-Chip. (I) zero-length spacer, (II) zero-length spacer on gelatin nanosubstrate, and (III) nm-PEG spacer on gelatin nanosubstrate. **(b)** Quantification of EVs captured on the surface of the microfluidic device for the different surface chemistries shown in (**a**) using confocal microscopy imaging. Ultimately, the combination of a nanostructure substrate and a nm-PEG linker on the surface of the microfluidic device maximized capture of tumor-EVs (III). For all experiments, Cetuximab was used as the capture antibody at 100 μg/mL (n =3 technical replicates; ± s.e.m., *, ** $p < 0.05$, 1-way ANOVA).

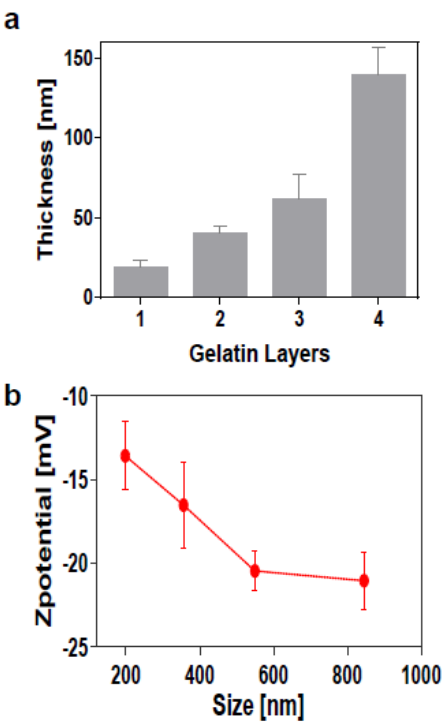

Supplementary Figure 5. (a) Thickness-growth curve of the gelatin nanocoating on a microfluidic substrate. **(b)** Change in the Zpotential of different populations of tumor-specific EVs (n = 3 technical replicates; ± s.e.m.).

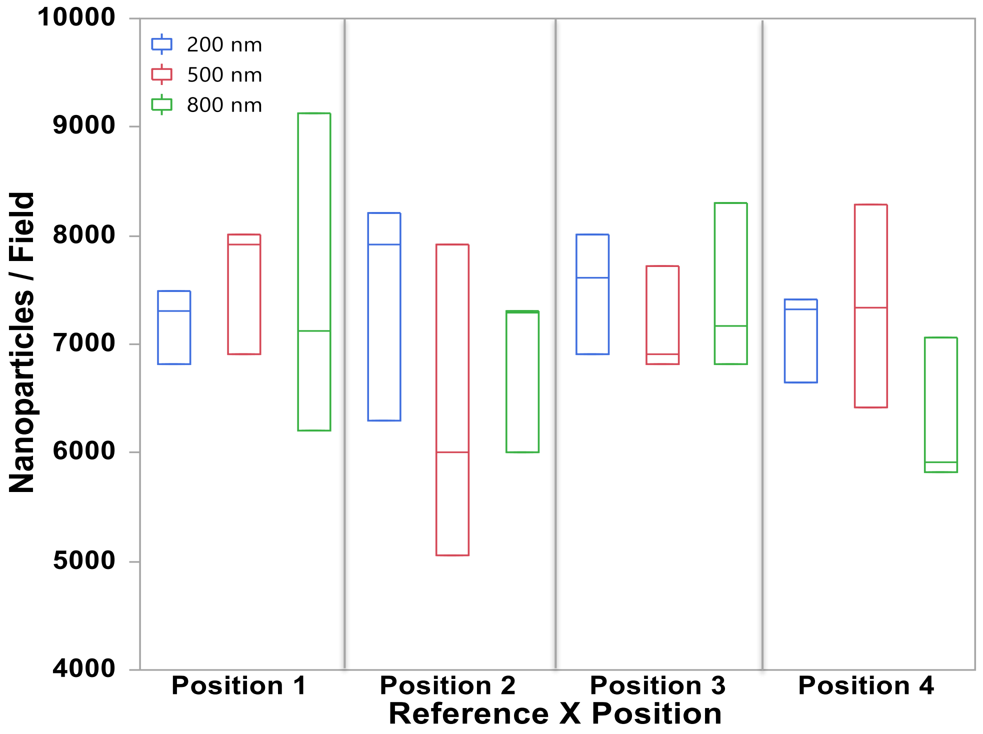

Supplementary Figure 6. Characterization of captured nanoparticle distribution across the surface of the microfluidic device. For each position, a 10x10 tile confocal microscopy (field) image was used and then quantified using Image J.

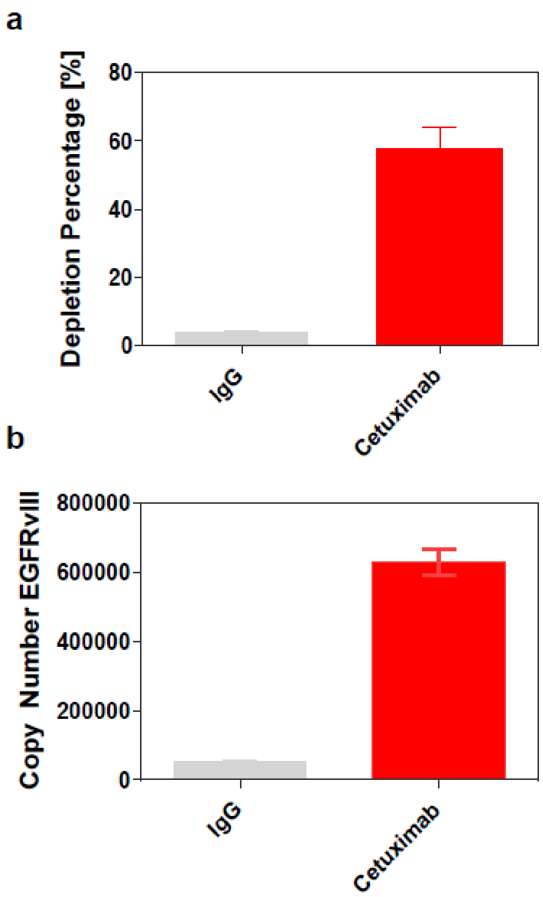

Supplementary Figure 7. (a) Tumor-EV depletion on the ^{EV}HB-Chip when using an irrelevant, IgG control antibody (gray) and Cetuximab (red). **(b)** Comparison of the specificity of capture for GFP Gli36 EVs for Cetuximab (red) and an IgG control antibody (gray) on the EVHB-Chip; ddPCR was used to quantify the total number of EGFRvIII copies. All experiments used EVs generated from GFP Gli36 parental cells (n = 3 technical replicates; ± s.e.m.).

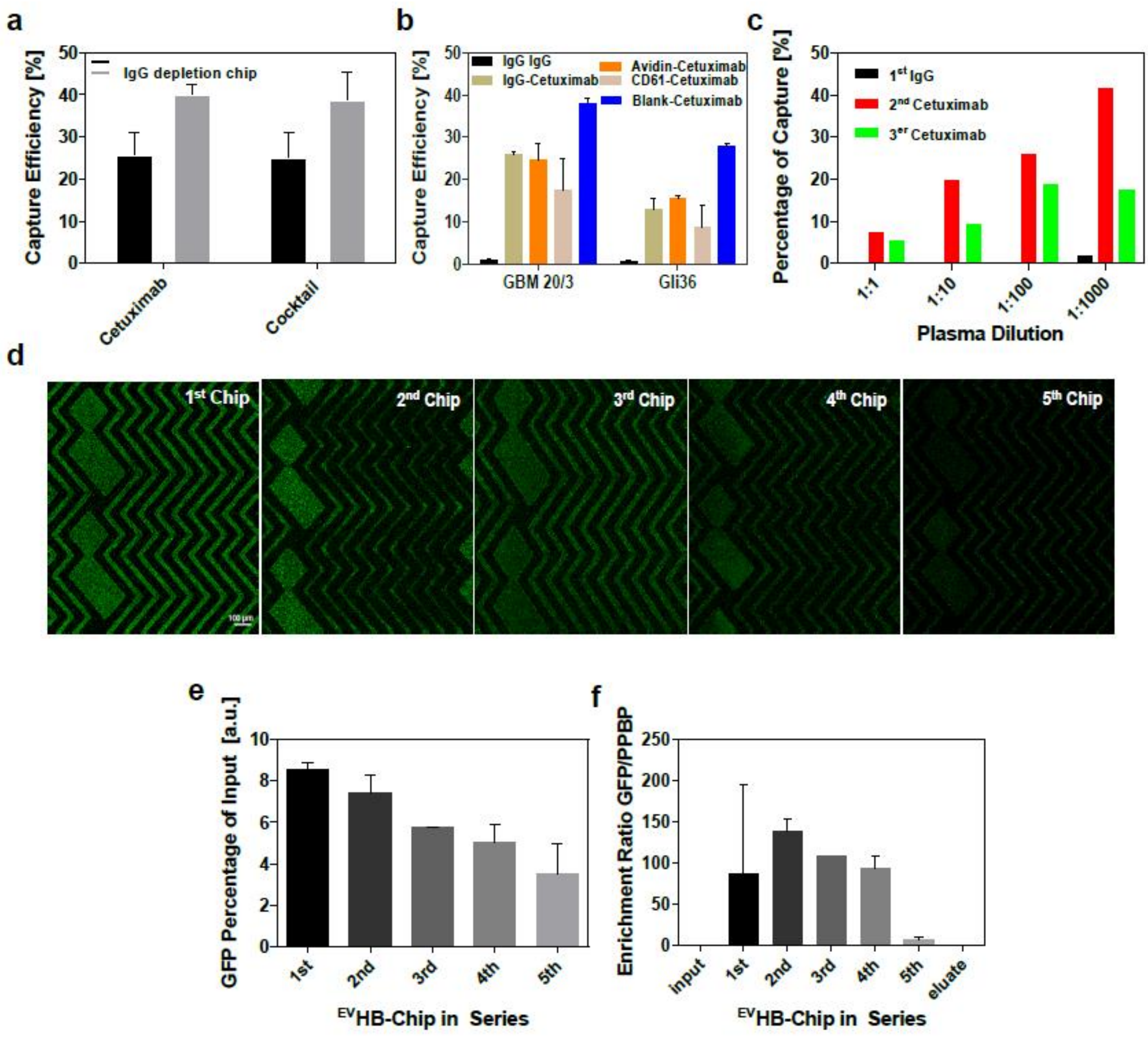

Supplementary Figure 8. (a) Comparison of the capture of GBM20/3 PalmGFP cells on a single ^{EV}HB-Chip or two chips run in series of which the first chip was coated with control IgG. The capture was analyzed by automated scanning of the second chip. (b) Capture efficiencies of GBM20/3 or Gli36 cells run through two chips in series. The first chip was coated with different control antibodies to analyze the effect on specific tumor cell capture in the second chip. Shown is the capture in the second ^{EV}HB-Chip analyzed using automated scanning. (c) Comparison of capture efficiencies of GBM 20/3-GFP EVs run through three ^{EV}HB-Chip in series. Multiple dilutions of the EV-plasma sample were tested for binding to the surface of the device. Captured GBM20/3-GFP EVs were lysed, RNA was isolated, and capture efficiency was analyzed using a TaqMan assay for GFP. (d,e,f). Experimental setup with five ^{EV}HB-Chip in series for the capture of tumor-specific GBM

20/3-GFP EVs. A blank chip was used in front of the functionalized chips. The capture was analyzed by confocal imaging (d), and RNA was analyzed for GFP (capture efficiency; e) and the GFP/PPBP ratio (enrichment levels) (f). A total of n = 3 technical replicates were performed.

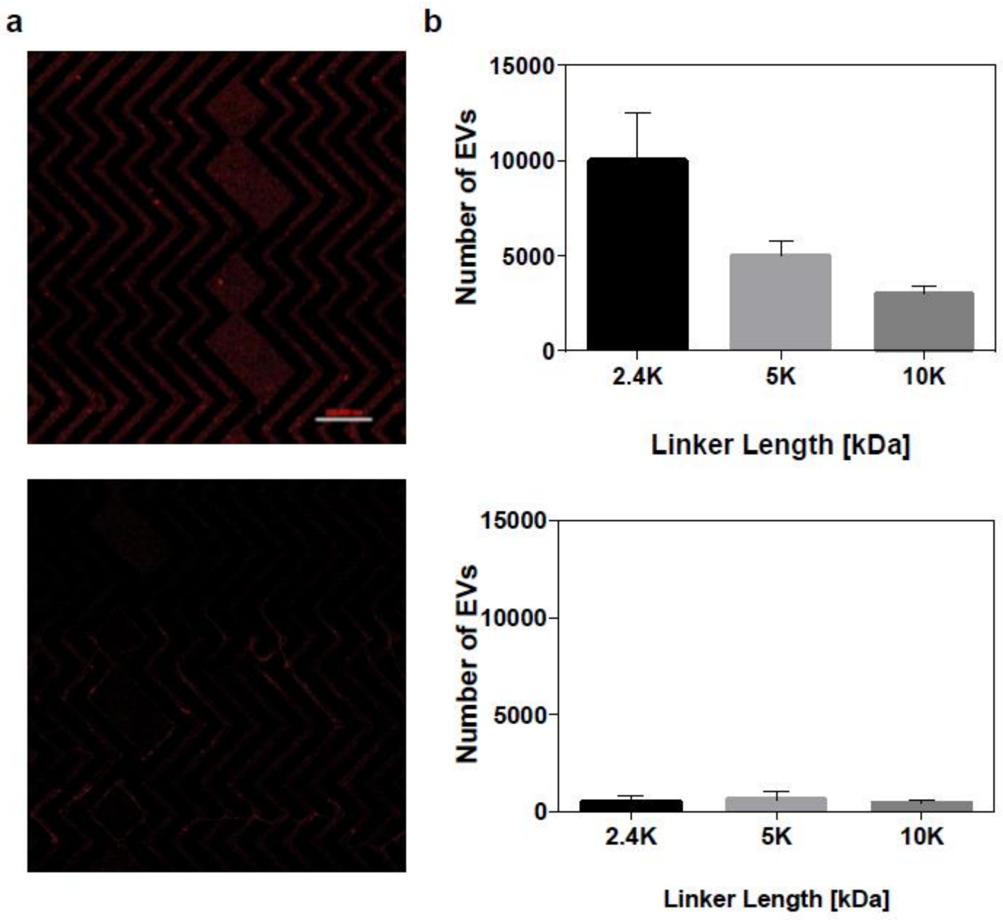

Supplementary Figure 9. Characterization of captured and released EVs from the surface of the ^{EV}HB-Chip. (a) Confocal fluorescence microscopy images before (top) and after (bottom) release of PalmtdTomato EVs by an increase of temperature from room temperature to 37°C. (b) Quantification of EV capture on the surface of the ^{EV}HB-Chip for different PEG-linker sizes. The bottom graph shows that over 87 % of captured EVs can be removed from the surface of the device (n = 3 technical replicates; ± s.e.m.).

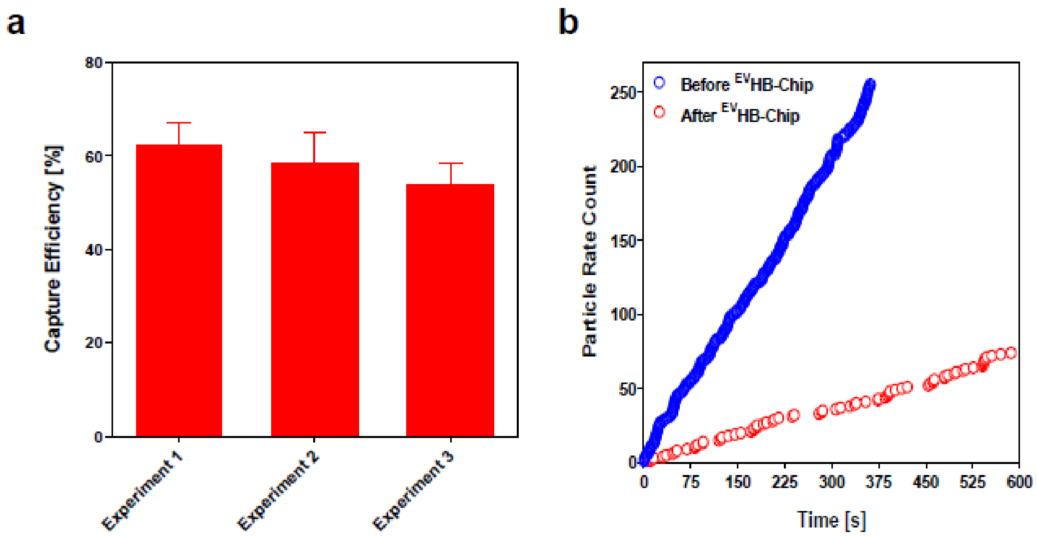

Supplementary Figure 10. **(a)** Capture efficiency on the ^{EV}HB-Chip for spiked TdTomato Glio36 EVs in PBS. (n = 3 technical replicates; ± s.e.m.; ^{EV}HB-Chip conditions used were the same as our patient samples). **(b)** Quantification of the depletion of EVs before and after it was run through the ^{EV}HB-Chip. The lower particle count after the sample was flow through the ^{EV}HB-Chip is an indication of EV depletion. The initial and final particle count of EVs was calculated using a qNano instrument before and after the samples were run through the ^{EV}HB-Chip.

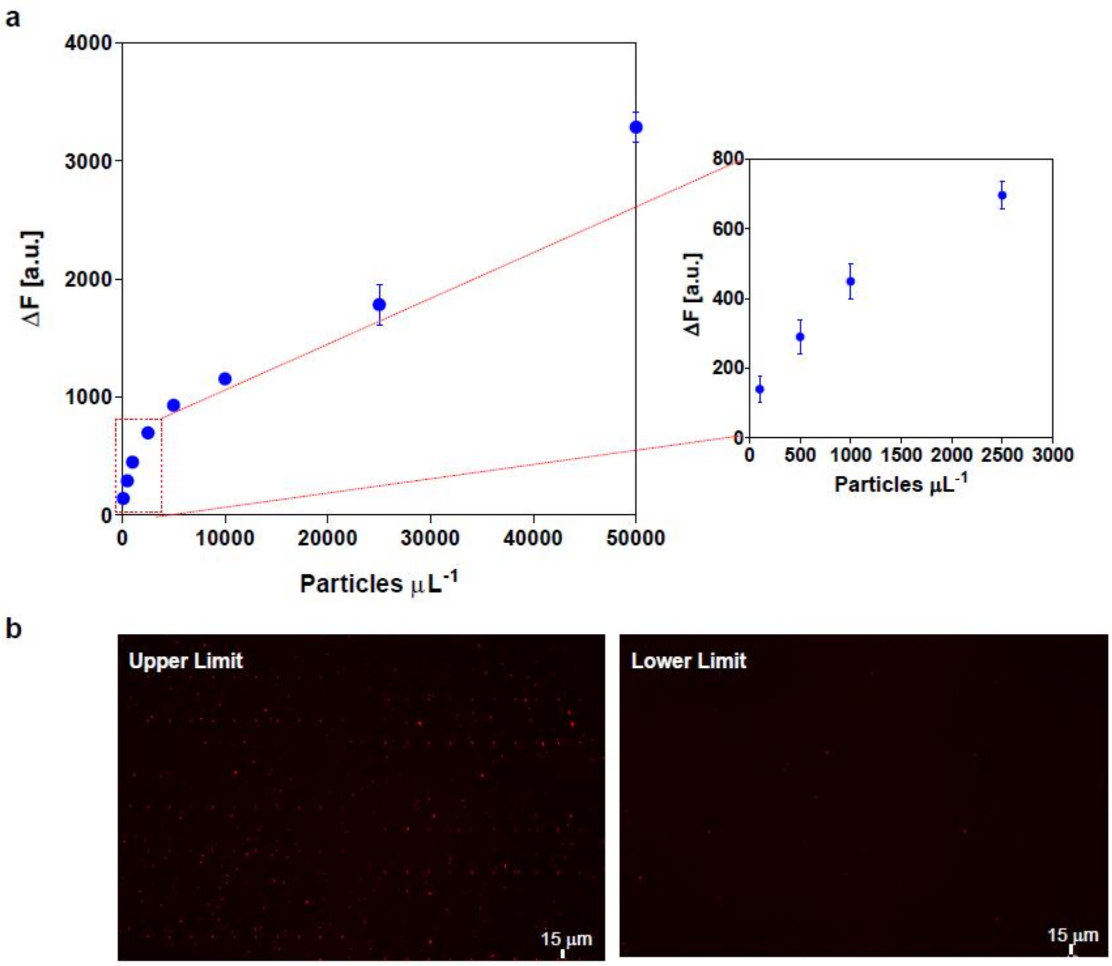

Supplementary Figure 11. The limit of detection of the EVHB-Chip by average fluorescence intensity of TdTomato Glio36 EVs. In **(a)** ΔF represents the change in fluorescence intensity between a blank device and a device that was run with different concentrations of EVs (Particles/ μL, n = 3 technical replicates; s.e.m.). **(b)** Typical images for the upper and lower limit of EV detection of the ^{EV}HB-Chip.

.

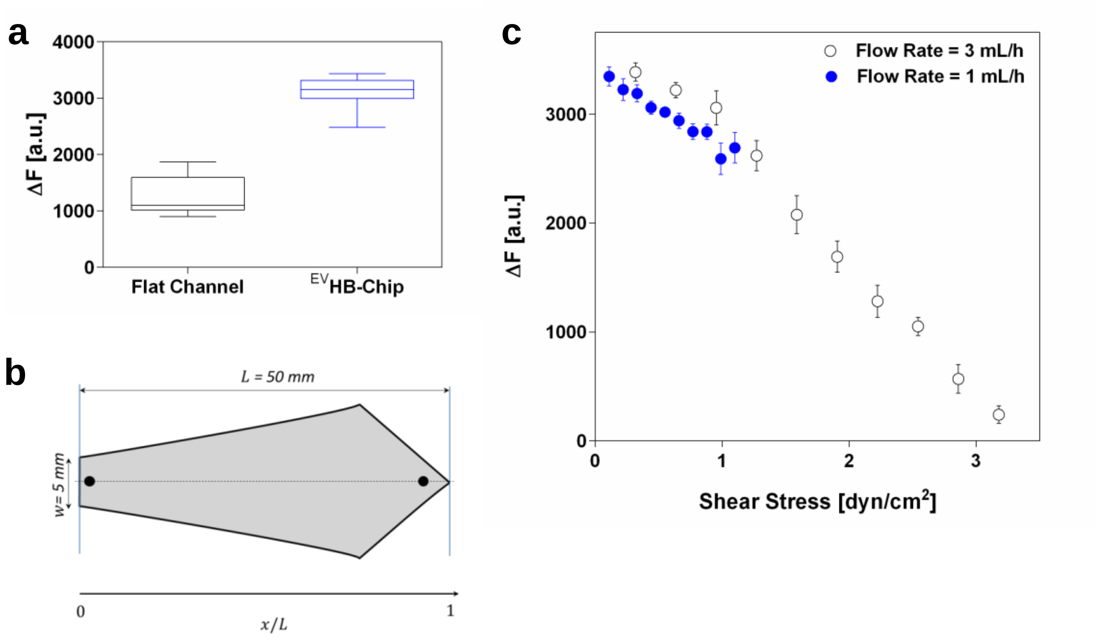

$$\tau_w = \frac{6\mu Q}{h^2 w}(1 - x/L)$$

Supplementary Figure 12. (a) Average fluorescence intensity measurement of EVs captured on a microfluidic flat channel and an ^{EV}HB-Chip. PalmtdTomato fluorescence EVs were spiked in PBS at 50 million EVs mL^{-1} (n = 3 technical replicates; ± s.e.m.). (b) Schematic representation of the Hele-Shaw device used to calculate the shear stress (τ_W) of EVs on microfluidic channels using the equation of Usami.[1] Where: *L* is the length in mm, *w* is the width in mm, *h* is the height in μm (for our case was set to 47 μm), μ is the viscosity of the fluid carrying the EVs, Q is the input flow rate, and x/L represents the normalized position at which the shear stress will be calculated. (c) Characterization of the shear stress in the Hele-Shaw device for two different flow rates (n = 3 technical replicates; ± s.e.m.). We performed experiments at two different flow rates (Q): At 1 mL h^{-1} (blue circles), the shear stress experienced by the EVs was 0.11 to 1.1 dyn cm^{-2} with a drop of 19.6 % in the fluorescence intensity of captured EVs. When the flow rate was 3 mL h^{-1} (open circles), the shear stress values were between 0.32 to 3.17 dyn cm^{-2} with a drop of 92.8 % in the fluorescence intensity of captured EVs. Therefore, we chose a flow rate of 1 mL h^{-1} to maximize specificity and EV capture.

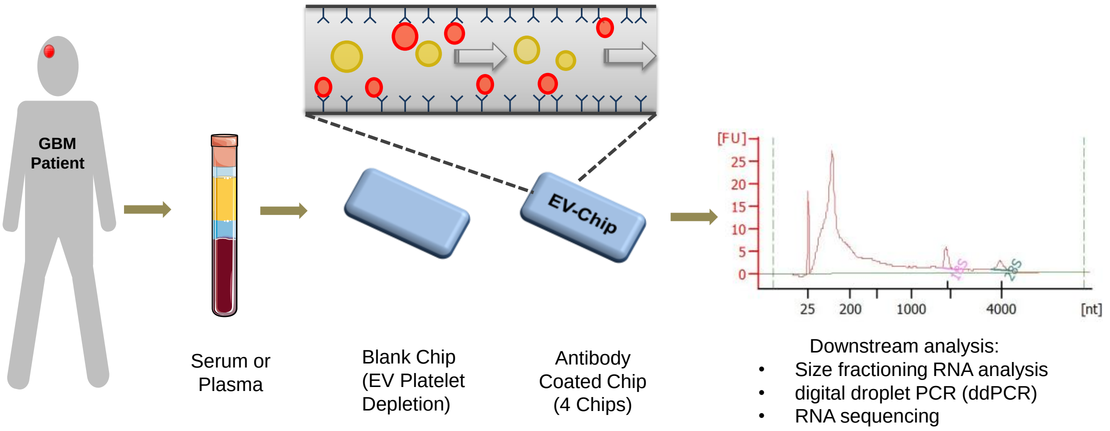

Supplementary Figure 13. Schematic representation of patient sample processing using the ^{EV}HB-Chip.

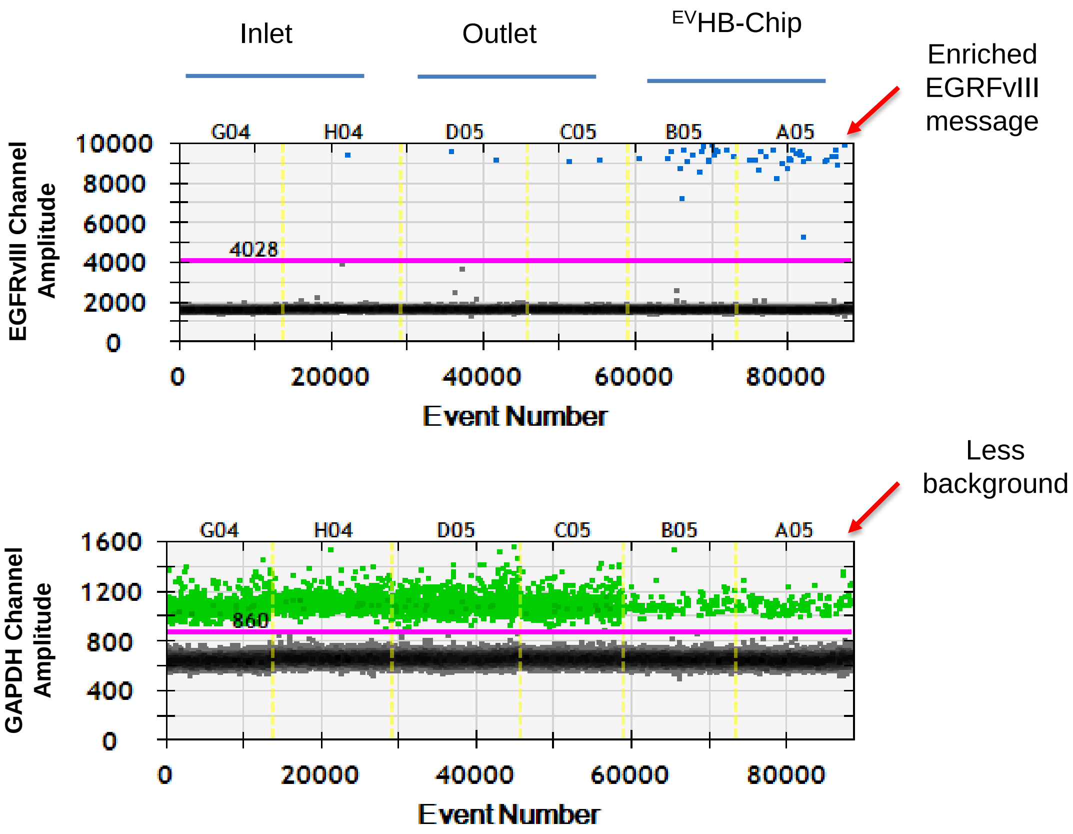

Supplementary Figure 14. Enrichment and specificity towards tumor-specific EVs (EGFRvIII). GAPDH was used as a control gene to demonstrate the specificity.

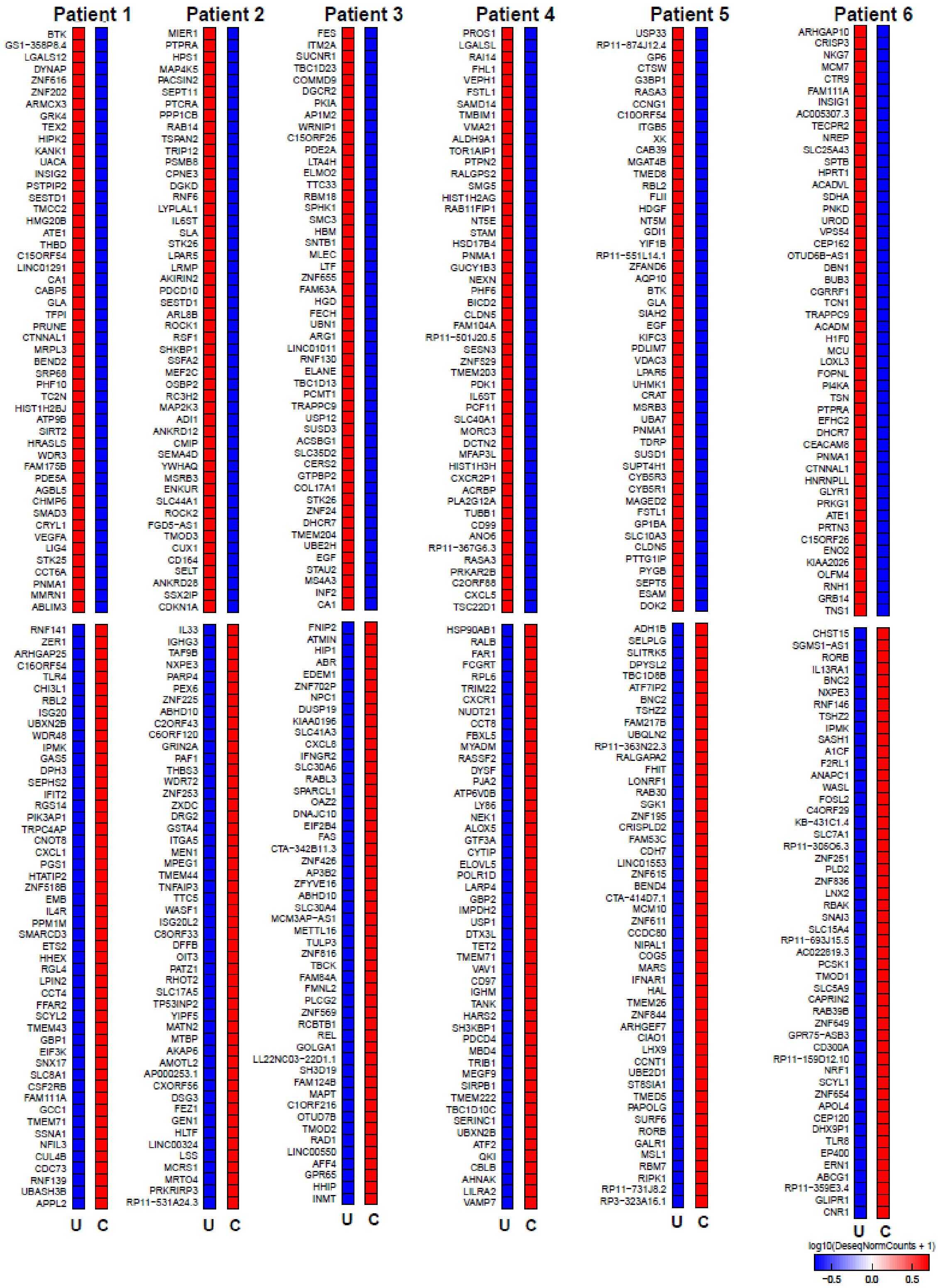

Supplementary Figure 15. Comparison of upregulated and downregulated genes between ultracentrifugation (U) and the ^{EV}HB-Chip (C) for each GBM patient.

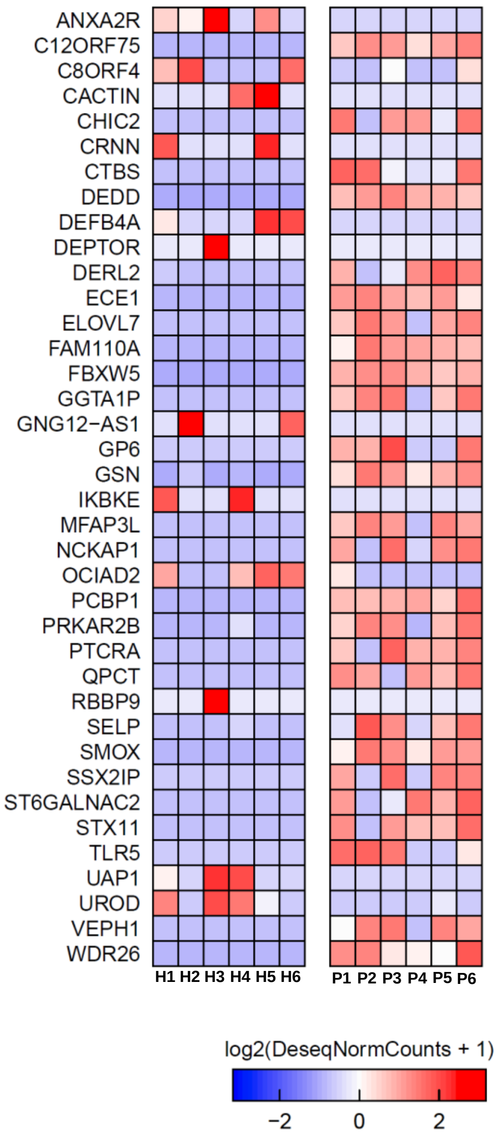

Supplementary Figure 16. Comparison of upregulated and downregulated genes in patients and healthy controls. The listed genes were previously identified in different types of cancers (see Supplementary Table 4).

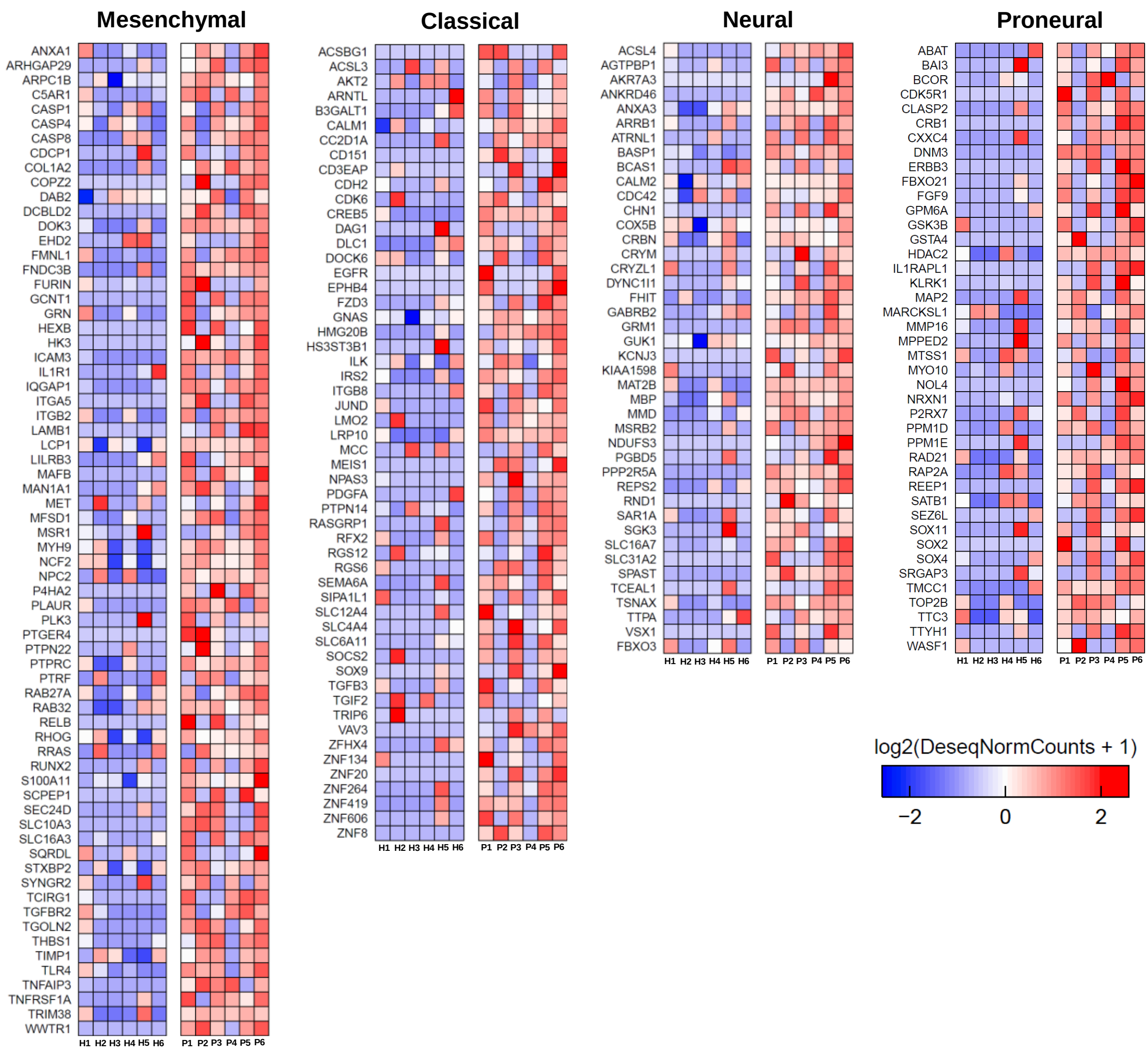

Supplementary Figure 17. Genes that were identified in EVs and categorized based on previously identified GBM subtype genes.

Supplementary Table 1. Clinical data for cancer patients.

	P1	P2	P3	P4	P5	P6	P7	P8	P9	P10	P11	P12	P13
**Age**	52	58	59	70	74	66	62	80	62	64	57	58	60
**Sex**	-	F	M	M	M	M	Male	Female	Male	Male	Male	Female	Female
**Location**	Brain Stem	L. Temporal	R. Frontal	L. Temporal	L. Temporal	L. Temporal	Temporal	Temporal	Parietal	Frontal, cingulate bilaterally	Temporal	Frontal	Frontotemporal
**Unifocal/Multifocal**	Unifocal	Unifocal	Unifocal	Unifocal	Unifocal	Unifocal	Unifocal	Unifocal	Unifocal	Multifocal	Unifocal	Unifocal	Unifocal
**Invades Callosum**	Yes	No	No	NTD	No	No	No	No	No	Yes	No	No	Yes
**Radiology**	Ring enhancing lesion	Cystic	Hemorrhagic	Ring enhancing lesion	Ring enhancing lesion	Cystic	Ring enhancing lesion	Ring enhancing lesion	Ring enhancing lesion	Ring enhancing lesion, Multifocal bilateral disease	Ring enhancing lesion	Patchy enhancement	Ring enhancement
**Ventricle involved**	Mass extends to it	No	-	NTD	No	No	Yes	Yes	Yes	Yes	No	Yes	Yes
**Prior hemorrhage**	Yes	NA	Possible	-	NTD	NTD	Yes	No	No	No	No	No	No
**Histology**	Glioblastoma	Glioblastoma	Glioblastoma (small cell)	Glioblastoma	Glioblastoma	Glioblastoma	Glioblastoma	Glioblastoma	Glioblastoma	Glioblastoma	Glioblastoma	Anaplastic Oligodendroglioma	Glioblastoma
**Mitosis**	Numerous	Numerous	Numerous	Yes	Yes	Yes	-	-	-	-	-	6.60%	-
**Necrosis**	Extensive	Extensive	Extensive	Extensive	Yes	-	Yes	Yes	Yes	Yes	Yes	No	Yes
**MIB1**	4 to 7 %	40%	NTD	30%	NTD	40%	-	-	-	-	-	6.60%	Yes
**IDH1**	Negative	Negative	NTD	Negative	NTD	Negative	WT	WT	WT	WT	WT	Mutant	No
**Tissue: EGFRvIII**	Yes	No	Yes	No	No	No	-	-	-	-	-	-	-
**CSF: EGFRvIII**	No	No	Yes	Not Tested	No	Yes	Not Tested	Not Tested	Not Tested	Not Tested	Not Tested	Not Tested	Not Tested
**Microfluidic Chip: EGFRvIII**	No	Yes	Yes	Yes	Yes	Yes	Not Tested	Not Tested	Not Tested	Not Tested	Not Tested	Not Tested	Not Tested
**Serum/Plasma**	Serum	Plasma	Plasma	Serum	Plasma	Plasma	Plasma	Plasma	Plasma	Plasma	Plasma	Plasma	Plasma
**Additional**	Negative for PDGFRa	Positive for GFAP	Negative for 1p/19q	Positive for EGFR	-	Negative for MGMT methylation; positive for EGFR	EGFR amplified, MGMT methylated, KRAS Mutant	MGMT methylated	EGFR amplified, MGMT methylated	BIOPSY (no resection) EGFR amplified	EGFR amplified	1p-19q co-deleted, p53 positive	MET amplified, MGMT methylated

Supplementary Table 2. RNA integrity number (RIN) and concentration values of first six GBM patients and healthy controls.

	Pt1	Pt2	Pt3	Pt4	Pt5	Pt6
RIN (from Chip)	1.2	2.4	1.4	1.8	2.3	2.2
RIN (unprocessed)	2.5	2.1	1.7	1	2.1	1.6
Concentration (from Chip) [pg/mL]	82	141	105	58	80	133
Concentration (unprocessed) [pg/mL]	142	84	45	34	37	68
	**H1**	**H2**	**H3**	**H4**	**H5**	**H6**
RIN (from Chip)	2.2	2.2	2.2	1	1.1	1.4
RIN (unprocessed)	1.6	2.2	2.4	1.1	1	1.1
Concentration (from Chip) [pg/mL]	111	86	669	35	45	52
Concentration (unprocessed) [pg/mL]	69	78	97	53	26	19

Supplementary Table 3. GBM specific genes differentially expressed genes in patient and healthy donor samples.

Gene	Function/Role in disease	Previously Identified in EVs	Reference
**ABCC3**	Encodes for transport proteins of different molecules in and out of the cell membrane. Involve in mechanisms of drug resistance.	N	2
**ACSL4**	Encodes for proteins that are involved in posttranslational modification for certain types of cancer. Overexpression of ACSL4 promotes tumor cell survival for preventing apoptosis.	N	3
**ACTN1**	Adhesion-related molecules enriched in the epithelial to mesenchymal transition signaling pathway.	N	4
**ALOX12**	Active ALOX12 protein affects development to skin tumor.	N	5
**AMFR**	High expression promotes in vitro migration and invasion of glioma cells	N	6
**ARHGEF7**	One of three genes of which its methylation signature allows the prediction of metastasis-free cancer and overall survival.	Y	7, 8
**ATP1B2**	Transcript of this gene is significantly lower in primary glioblastomas than in diffuse astrocytomas.	N	9
**ATP2A3**	Cancer susceptibility.	N	10
**BASP1**	This gene is upregulated in GBM after SOX2 knockdown.	Y	11
**CD151**	The protein encoded by this gene is a member of the transmembrane 4 superfamily, also known as the tetraspanin family. Most of these members are cell-surface proteins that are characterized by the presence of four hydrophobic domains. The proteins mediate signal transduction events that play a role in the regulation of cell development, activation, growth, and motility. This encoded protein is a cell surface glycoprotein that is known to complex with integrins and other transmembrane 4 superfamily proteins. It is involved in cellular processes including cell adhesion and may regulate integrin trafficking and/or function. This protein enhances cell motility, invasion, and	N	12, 13

	metastasis of cancer cells. Multiple alternatively spliced transcript variants that encode the same protein have been described for this gene. Overexpression of CD151 affects cancer invasion and metastasis.		
**CDC14B**	Acts upstream of skp2/p21/p27 pathway, promoting skP2 degradation through Ser64 dephosphorylation.	N	14
**CDKN1A**	This gene encodes a potent cyclin-dependent kinase inhibitor. The encoded protein binds to and inhibits the activity of cyclin-cyclin-dependent kinase2 or -cyclin-dependent kinase4 complexes, and thus functions as a regulator of cell cycle progression at G1. The expression of this gene is tightly controlled by the tumor suppressor protein p53, through which this protein mediates the p53-dependent cell cycle G1 phase arrest in response to a variety of stress stimuli. This protein can interact with proliferating cell nuclear antigen, a DNA polymerase accessory factor, and plays a regulatory role in S phase DNA replication and DNA damage repair. This protein was reported to be specifically cleaved by CASP3-like caspases, which thus leads to a dramatic activation of cyclin-dependent kinase2, and may be instrumental in the execution of apoptosis following caspase activation. Mice that lack this gene can regenerate damaged or missing tissue. Multiple alternatively spliced variants have been found for this gene.	N	15
**CEP19**	The protein encoded by this gene localizes to centrosomes and primary cilia and co-localizes with a marker for the mother centriole. Morbid obesity results from the inactivation of the ciliary protein CEP19.	N	16, 17
**CLDN5**	This gene encodes a member of the claudin family. Claudins are integral membrane proteins and components of tight junction strands. Has a possible correlation with Glioma tumor grade.	N	18
**CLEC2L**	CLEC2L may be involved in the cellular cross-talk of Purkinje cells and other neurons with brain-resident cells or brain-infiltrating immune cells.	N	19

**CREG2**	CREG is a secreted glycoprotein that enhances differentiation of pluripotent stem cells.	N	20
**CXCL5**	This protein is thought to play a role in cancer cell proliferation, migration, and invasion.	N	21
**E2F3**	E2F3 is the dominant effector of miR-195-mediated cell cycle arrest. High levels of E2F3 protein activate the transcription of genes promoting cell cycle progression, which leads to abnormal cell proliferation.	N	22
**EMHT2**	Reportedly involved with important cancer-sustaining cellular activities such as cell proliferation, autophagy, EMT, metabolic changes, specific responses to hypoxia and cancer stemness	N	23
**EI24**	Etoposide-induced gene 24 (Ei24) is a p53 target gene that inhibits growth, induces apoptosis and autophagy, as well as suppresses breast cancer.	N	24
**GADD45A**	Gadd45 genes have been implicated in stress signaling in response to physiological or environmental stressors, which results in cell cycle arrest, DNA repair, cell survival, and senescence, or apoptosis.	N	25
**GAS2L1**	Members of the GAS2 family mediates the crosstalk between filamentous actin (F-actin) and microtubules which have an important role in cell polarization and cell motility	N	26
**GJC1**	This gene is a member of the connexin gene family and a component of a gap junction. Acts as a tumor suppressor in most cancer cells.	N	27
**GRIA1**	Marker for Glioma-initiating cells, and potentially a marker for predicting Ca2+ sensitivity of the cells.	N	28
**GUCY1A3**	This gene encodes the alpha subunit of the soluble guanylate cyclase (sGC), which catalyzes the conversion of GTP (guanosine triphosphate) to cGMP (cyclic guanosine monophosphate). sGC interacts with Nitric Oxide and has a role in the induction of angiogenesis in human glioma cell lines.	N	29
**GUCY1B3**	This gene encodes the beta subunit of the soluble guanylate cyclase (sGC), which catalyzes the conversion of GTP (guanosine triphosphate) to cGMP (cyclic guanosine monophosphate). sGC interacts with Nitric Oxide and has a	N	29

	role in the induction of angiogenesis in human glioma cell lines.		
**ID1**	ID proteins, which are cell fate determinants, are involved in a broad range of processes associated with tumorigenesis. For example, ID proteins promote cell proliferation by suppressing cell-cycle–negative regulators.	N	30
**ID3**	ID proteins, which are cell fate determinants, are involved in a broad range of processes associated with tumorigenesis. For example, ID proteins promote cell proliferation by suppressing cell-cycle–negative regulators	N	30
**IRAK3**	Exerts an overall inhibitory effect on inflammatory response. Also, inhibits TLR7-mediated production of cytokines and chemokines at translational levels.	N	31
**ITGB3**	Participate in cell adhesion as well as cell-surface mediated signaling. Also, ITGB3 is implicated in glioma cell death/survival and drug resistance.	N	32
**KBTBD7**	Cullin 3 (Cul3)/KBTBD7 complex controls both the regulated proteasomal degradation of neurofibromin(NF1) and the pathogenic destabilization of neurofibromin in glioblastomas.	N	33
**KIAA0513**	KIAA0513 is likely to be involved in signaling pathways related to neuroplasticity, apoptosis, and cytoskeletal regulation	N	34
**KLHL2**	KLHL12 has a role in oligodendrocyte process formation. Also associated with ubiquitylation, aggregation and neuronal apoptosis.	N	35
**KRAS**	RAS proteins functions in cellular signal transduction. They influence cellular growth, differentiation, and survival.	N	36
**LCN2**	Increases the stability of MMP-9. Transports small hydrophobic molecules. Also, influences iron transport and storage.	N	37
**LRRTM2**	A key regulator of excitatory synapse development and function of neurons.	N	38
**LTBP1**	Regulates secretion and activation of TGF-betas.	N	39
**MAP3K1**	The mutant version of this gene is associated with GBM and breast adenocarcinomas as well as head and neck squamous carcinoma.	N	40, 41
**MAST3**	This gene has a regulatory role on PTEN function by controlling its stability and phosphorylation status.	N	42

**METTL15**	The Mutant form is observed with adenocarcinoma and endometrioid carcinomas.	N	43
**MLLT11**	MLLT11 specifically binds to T-cell-factor-7 (TCF7) in the Wnt signaling pathway and results in transcriptional activation of CD44	N	44
**NSF**	NSF is a cytoplasmic ATPase that disassembles the SNARE complexes.	N	45
**NUCB1**	EF-hand containing calcium-binding protein and a guanine nucleotide exchange factor for trimeric G protein, Giα that is required for unfolded protein response. The role of its GEF function remains unknown.	N	46
**OCIAD2**	Down-regulated in Proneural GBM	N	47
**P2RY12**	P2Y12 regulates platelet adhesion/activation, thrombus growth, and thrombus stability in injured arteries.	N	48
**PEX5L**	Mutant version observed in adenocarcinoma, embryonal rhabdomyosarcoma, endometrioid carcinoma, melanoma.	N	49, 50
**PIK3CA**	Catalytic subunit of Phosphatidylinositol 3-kinase. (PI3K) are a family of lipid kinases involved in diverse cellular signaling pathways, including proliferation, differentiation, migration, trafficking, and glucose homeostasis.	N	51
**PRKAR1B**	PKA stimulates the expression of the NR4 receptor, and NR4 is involved in several malignancies, such as glioblastoma.	N	52
**PRKY**	Pseudogene, target for nonsense-mediated decay.	N	
**PROS1**	Glycoprotein involved in cell adhesion.	N	53
**PYGB**	The protein encoded by this gene is a glycogen phosphorylase found predominantly in the brain. The activity of this enzyme is positively regulated by AMP and negatively regulated by ATP, ADP, and glucose-6-phosphate. This enzyme catalyzes the rate-determining step in glycogen degradation. The mutant form is observed with adenocarcinoma, squamous-cell carcinoma, adenocarcinoma and clear-cell adenocarcinoma.	N	43, 54, 55
**SP3**	Bifunctional transcription factor. Activating Metalloproteinase 2 expression in astroglioma cells.	N	56

**STAT5B**	Promotes oncogenesis and treatment resistance in glioblastoma by direct regulation of anti-apoptotic gene, Bcl-XL.	N	57
**TMEM106C**	The upregulation of this gene is associated with clinical stage III breast cancer.	N	58, 59
**VAMP7**	SNARE family transmembrane transport protein.Mediates secretory lysosome exocytosis, contributing to release of both ATP and cathepsin B from glial cells.	N	60

Supplementary Table 4. Cancer genes differentially expressed genes in patient and healthy donor samples.

Gene	Function	Previuosly Identified in EVs	Reference
**ANXA2R**	Up-regulation promotes proliferation of breast cancer cells; promotes hepatocellular cancer cell invasion and migration in vitro.	N	61, 62
**ATP2A3**	Overexpression indicates cancer susceptibility.	Y	63
**C8orf4**	Overexpression promotes proliferation of non-small cell lung carcinoma; high expression in ovarian carcinomas; high expression in 50 % of breast cancers; high expression in gastric cancers; down-regulated in metastatic colon cancer; overexpressed in thyroid carcinoma.	N	64-69
**C12orf75**	Overexpressed in colon carcinoma.	N	70
**CACTIN**	Present in spindle cell type/renal cell carcinoma.	N	71
**CHIC2**	In some cases of acute myeloid leukemia found recombined to ETV-6 gene.	N	72
**CRNN**	Altered expression in laryngo-pharyngeal tumors; down-regulated in esophageal squamous cell carcinoma; present in head and neck squamous cell carcinoma.	N	73-75
**CTBS**	CTBS fusions seen in breast and ovarian cancer cell lines.	N	76
**DEDD**	The expression is conversely related to poor prognosis in breast and colon cancer.	N	77
**DEFB4A**	Lower copy numbers are associated with susceptibility to cervical cancer; down-regulated in colon cancer; up-regulated in squamous and basal skin cell carcinoma.	N	78-80
**DEPTOR**	Down-regulated in pancreatic, colorectal cancer; up-regulated in differentiated thyroid carcinoma; down-regulated in basal-like/triple negative breast cancer; overexpressed in multiple myelomas.	N	81-85
**DERL2**	Up-regulated in liver cancer.	N	86

**ECE1**	Up-regulated in gastric cancers; up-regulation in breast cancer reflects worse prognosis; up-regulated in malignant prostate cancer.	N	87-89
**ELOVL7**	Overexpressed in prostate cancer and required for prostate cancer-cell growth.	N	90
**FAM110A**	Mesenchymal marker in non-small cell lung carcinoma.	N	91
**FBXW5**	Up-regulated in non-small cell lung carcinoma.	N	92
**GGTA1P**	Expression can cause germ cell tumors.	N	93
**GNG12-AS1**	Down-regulated in breast cancer.	N	94
**GP6**	This gene encodes a platelet membrane glycoprotein of the immunoglobulin superfamily. The encoded protein is a receptor for collagen and plays a critical role in collagen-induced platelet aggregation and thrombus formation.	N	95
**GSN**	Down-regulated in breast pancreatic, and bladder cancer.	N	96-99
**IKBKE**	Breast and prostate cancer oncogene; up-regulated in squamous lung cell carcinoma; promotes metastasis in ovarian cancer.	N	100-103
**MFAP3L**	Activation promotes colorectal cancer cell invasion.	N	104
**NCKAP1**	Variations of expression are associated with poor prognosis in breast cancer patients.	N	105
**OCIAD2**	Highly expressed in bronchoalveolar carcinoma; highly expressed in ovarian cancers.	N	106, 107
**PCBP1**	Essential for cancer stem cells of prostate cancer; essential for epithelial-mesenchymal transition in gastric cancer.	N	108, 109
**PRKAR2B**	Down-regulated in cholangiocarcinoma.	N	110
**PTCRA**	Essential for T-cell leukemogenesis in T-cell lymphoblastic leukemia.	N	111
**QPCT**	Up-regulated in thyroid carcinoma.	N	112
**RBBP9**	Elevated activity in pancreatic cancer.	N	113
**SELP**	Expressed on activated endothelial cells and platelets; mediates adhesion and seeding of cancer cells.	N	114
**SMOX**	Down-regulated in breast cancer; up-regulated in prostate cancer.	N	115, 116
**SSX2IP**	Up-regulated in hepatocellular carcinoma; upregulated in melanoma, colon, and breast	N	117-119

	cancer; upregulated in acute myeloid leukemia.		
**ST6GALNAC2**	High expression in mammary phyllodes tumor; reflects tumorigenicity.	N	120
**STX11**	Acts as a tumor suppressor in peripheral T-cell lymphomas.	N	121
**TLR5**	Up-regulated in non-small cell lung carcinoma; upregulated in squamous cell carcinoma of the tongue; command TLR5 mutations in cancer progression.	N	122-124
**UAP1**	Overexpressed in prostate cancer; amplified in liposarcoma.	N	125, 126
**UROD**	Overexpressed in head and neck cancer.	N	127
**VEPH1**	Potential effect on ovarian cancer.	N	128
**WDR26**	Promotes breast cancer.	N	129, 130

Supplementary References

1. Usami, S., Chen, H.-H., Zhao, Y., Chien, S. & Skalak, R. Design and construction of a linear shear stress flow chamber. *Ann. Biomed. Eng.* **21**, 77-83 (1993).
2. Huang, Y. et al. Membrane Transporters and Channels. *Role of the Transportome in Cancer Chemosensitivity and Chemoresistance* **64**, 4294-4301 (2004).
3. Currie, E., Schulze, A., Zechner, R., Walther, Tobias C. & Farese Jr, Robert V. Cellular Fatty Acid Metabolism and Cancer. *Cell Metabolism* **18**, 153-161 (2013).
4. Meng, J., Li, P., Zhang, Q., Yang, Z. & Fu, S. A radiosensitivity gene signature in predicting glioma prognostic via EMT pathway. (2014).
5. S, K. & HE, B. Significance of lipid mediators in corneal injury and repair. *J Lipid Res* **51**, 879-891. Epub 2009 Nov 2003 (2009).
6. Choudhury, Y. et al. Attenuated adenosine-to-inosine editing of microRNA-376a* promotes invasiveness of glioblastoma cells. *The Journal of Clinical Investigation* **122**, 4059-4076.
7. Heyn, H. & Esteller, M. DNA methylation profiling in the clinic: applications and challenges. *Nat Rev Genet* **13**, 679-692 (2012).
8. Fortin Ensign, S., Mathews, I., Symons, M., Berens, M. & Tran, N. Implications of Rho GTPase Signaling in Glioma Cell Invasion and Tumor Progression. *Frontiers in Oncology* **3** (2013).
9. van den Boom, J., Wolter, M., Blaschke, B., Knobbe, C.B. & Reifenberger, G. Identification of novel genes associated with astrocytoma progression using suppression subtractive hybridization and real-time reverse transcription-polymerase chain reaction. *Int. J. Cancer* **119**, 2330-2338 (2006).
10. Korošec, B., Glavač, D., Volavšek, M. & Ravnik-Glavač, M. ATP2A3 gene is involved in cancer susceptibility. *Cancer Genet. Cytogenet.* **188**, 88-94 (2009).
11. Fang, X. et al. The SOX2 response program in glioblastoma multiforme: an integrated ChIP-seq, expression microarray, and microRNA analysis. *BMC Genomics* **12**, 11 (2011).

12. NM_004357.4, R.A.
13. Lee, D. et al. Prognostic significance of tetraspanin CD151 in newly diagnosed glioblastomas. *Journal of Surgical Oncology* **107**, 646-652 (2013).
14. Galeano, F. et al. ADAR2-editing activity inhibits glioblastoma growth through the modulation of the CDC14B/Skp2/p21/p27 axis. *Oncogene* **32**, 998-1009 (2013).
15. NG_009364.1, R.A.
16. Jakobsen, L. et al. Novel asymmetrically localizing components of human centrosomes identified by complementary proteomics methods. *The EMBO Journal* **30**, 1520-1535 (2011).
17. Shalata, A. et al. Morbid Obesity Resulting from Inactivation of the Ciliary Protein CEP19 in Humans and Mice. *Am. J. Hum. Genet.* **93**, 1061-1071 (2013).
18. Hanuma, K.K. et al. Down Regulated Expression of Claudin-1 and Claudin-5 and Up Regulation of β-Catenin: Association with Human Glioma Progression. *CNS & Neurological Disorders - Drug Targets* **13**, 1413-1426 (2014).
19. Lysenko, O., Schulte, D., Mittelbronn, M. & Steinle, A. BACL Is a Novel Brain-Associated, Non-NKC-Encoded Mammalian C-Type Lectin-Like Receptor of the CLEC2 Family. *PloS One* **8**, e65345 (2013).
20. Kunita, R., Otomo, A. & Ikeda, J.-E. Identification and Characterization of Novel Members of the CREG Family, Putative Secreted Glycoproteins Expressed Specifically in Brain. *Genomics* **80**, 456-460 (2002).
21. Xu, X., Huang, P., Yang, B., Wang, X. & Xia, J. Roles of CXCL5 on migration and invasion of liver cancer cells. *J. Transl. Med.* **12**, 1-11 (2014).
22. Zhang, Q.-Q. et al. MicroRNA-195 plays a tumor-suppressor role in human glioblastoma cells by targeting signaling pathways involved in cellular proliferation and invasion. *Neuro-Oncology* **14**, 278-287 (2012).

23. Ding, J. et al. The Histone H3 Methyltransferase G9A Epigenetically Activates the Serine-Glycine Synthesis Pathway to Sustain Cancer Cell Survival and Proliferation. *Cell Metabolism* **18**, 896-907.
24. Devkota, S. et al. Ei24-deficiency attenuates protein kinase Cα signaling and skin carcinogenesis in mice. *The International Journal of Biochemistry & Cell Biology* **44**, 1887-1896 (2012).
25. Liebermann, D.A. & Hoffman, B. Gadd45 in stress signaling. *Journal of Molecular Signaling* **3**, 15-15 (2008).
26. Goriounov, D., Leung, C.L. & Liem, R.K.H. Protein products of human related genes on chromosomes 17 and 22 associate with both microfilaments and microtubules. *J. Cell Sci.* **116**, 1045-1058 (2003).
27. King, T.J. & Bertram, J.S. Connexins as targets for cancer chemoprevention and chemotherapy. *Biochimica et Biophysica Acta (BBA) - Biomembranes* **1719**, 146-160 (2005).
28. Wee, S. et al. Selective Calcium Sensitivity in Immature Glioma Cancer Stem Cells. *PloS One* **9**, e115698 (2014).
29. Saino, M., Maruyama, T., Sekiya, T., Kayama, T. & Murakami, Y. Inhibition of angiogenesis in human glioma cell lines by antisense RNA from the soluble guanylate cyclase genes, GUCY1A3 and GUCY1B3. *Oncol. Rep.* **12**, 47 - 52 (2004).
30. Alani, R.M., Young, A.Z. & Shifflett, C.B. Id1 regulation of cellular senescence through transcriptional repression of p16/Ink4a. *Proc. Natl. Acad. Sci.* **98**, 7812-7816 (2001).
31. Zhou, H. et al. IRAK-M mediates Toll-like receptor/IL-1R-induced NFκB activation and cytokine production. *The EMBO Journal* **32**, 583-596 (2013).
32. Kim, J.-H., Zheng, L.T., Lee, W.-H. & Suk, K. Pro-apoptotic role of integrin β3 in glioma cells. *J. Neurochem.* **117**, 494-503 (2011).
33. Hollstein, P.E. & Cichowski, K. Identifying the ubiquitin ligase complex that regulates the NF1 tumor suppressor and Ras. *Cancer Discovery* **3**, 10.1158/2159-8290.CD-1113-0146 (2013).

34. Lauriat, T.L. et al. Characterization of KIAA0513, a novel signaling molecule that interacts with modulators of neuroplasticity, apoptosis, and the cytoskeleton. *Brain Res.* **1121**, 1-11 (2006).
35. Tseng, L.A. & Bixby, J.L. Interaction of an Intracellular Pentraxin with a BTB-Kelch Protein is Associated with Ubiquitylation, Aggregation and Neuronal Apoptosis. *Molecular and cellular neurosciences* **47**, 254-264 (2011).
36. Goodsell, D.S. The Molecular Perspective: The ras Oncogene. *The Oncologist* **4**, 263-264 (1999).
37. Candido, S. et al. Roles of NGAL and MMP-9 in the tumor microenvironment and sensitivity to targeted therapy. *Biochimica et Biophysica Acta (BBA) - Molecular Cell Research* **1863**, 438-448 (2016).
38. de Wit, J. et al. LRRTM2 Interacts with Neurexin1 and Regulates Excitatory Synapse Formation. *Neuron* **64**, 799-806 (2009).
39. Beaufort, N. et al. Cerebral small vessel disease-related protease HtrA1 processes latent TGF-β binding protein 1 and facilitates TGF-β signaling. *Proceedings of the National Academy of Sciences of the United States of America* **111**, 16496-16501 (2014).
40. C, K. et al. Mutational landscape and significance across 12 major cancer types. *Nature* **502**, 333-339 (2013).
41. O'Leary, N.A. et al. Reference sequence (RefSeq) database at NCBI: current status, taxonomic expansion, and functional annotation. *Nucleic Acids Res.* **44**, D733-D745 (2016).
42. Valiente, M. et al. Binding of PTEN to Specific PDZ Domains Contributes to PTEN Protein Stability and Phosphorylation by Microtubule-associated Serine/Threonine Kinases. *J. Biol. Chem.* **280**, 28936-28943 (2005).
43. Kumar, A. et al. Substantial inter-individual and limited intra-individual genomic diversity among tumors from men with metastatic prostate cancer. *Nat. Med.* **22**, 369-378 (2016).

44. Park, J. et al. AF1q is a novel TCF7 co-factor which activates CD44 and promotes breast cancer metastasis. *Oncotarget* **6**, 20697-20710 (2015).

45. Puri, N., Kruhlak, M.J., Whiteheart, S.W. & Roche, P.A. Mast Cell Degranulation Requires N-Ethylmaleimide-Sensitive Factor-Mediated SNARE Disassembly. *The Journal of Immunology* **171**, 5345-5352 (2003).

46. Barbazan, J. et al. Prognostic Impact of Modulators of G proteins in Circulating Tumor Cells from Patients with Metastatic Colorectal Cancer. *Scientific Reports* **6**, 22112 (2016).

47. Noushmehr, H. et al. Identification of a CpG Island Methylator Phenotype that Defines a Distinct Subgroup of Glioma. *Cancer Cell* **17**, 510-522 (2010).

48. André, P. et al. P2Y︵12︶ regulates platelet adhesion/activation, thrombus growth, and thrombus stability in injured arteries. *J. Clin. Invest.* **112**, 398-406 (2003).

49. van de Wetering, M. et al. Prospective Derivation of a Living Organoid Biobank of Colorectal Cancer Patients. *Cell* **161**, 933-945.

50. Krauthammer, M. et al. Exome sequencing identifies recurrent mutations in NF1 and RASopathy genes in sun-exposed melanomas. *Nat. Genet.* **47**, 996-1002 (2015).

51. Gallia, G.L. et al. PIK3CA Gene Mutations in Pediatric and Adult Glioblastoma Multiforme. *Mol. Cancer Res.* **4**, 709-714 (2006).

52. Wong, T.H. et al. PRKAR1B mutation associated with a new neurodegenerative disorder with unique pathology. *Brain* **137**, 1361-1373 (2014).

53. Milinkovic, V. et al. Identification of Novel Genetic Alterations in Samples of Malignant Glioma Patients. *PloS One* **8**, e82108 (2013).

54. Mouradov, D. et al. Colorectal Cancer Cell Lines Are Representative Models of the Main Molecular Subtypes of Primary Cancer. *Cancer Res.* **74**, 3238-3247 (2014).

55. Martin, D. et al. The head and neck cancer cell oncogenome: a platform for the development of precision molecular therapies. *Oncotarget* **5**, 8906-8923 (2014).
56. Qin, H., Sun, Y. & Benveniste, E.N. The Transcription Factors Sp1, Sp3, and AP-2 Are Required for Constitutive Matrix Metalloproteinase-2 Gene Expression in Astroglioma Cells. *J. Biol. Chem.* **274**, 29130-29137 (1999).
57. Latha, K. et al. Nuclear EGFRvIII-STAT5b complex contributes to glioblastoma cell survival by direct activation of the Bcl-XL promoter. *International journal of cancer. Journal international du cancer* **132**, 509-520 (2013).
58. MA, F. et al. Gene expression profiling of clinical stages II and III breast cancer. *Braz J Med Biol Res* **39**, 1101-1113 (2006).
59. A, N. et al. BEX2 is overexpressed in a subset of primary breast cancers and mediates nerve growth factor/nuclear factor-kappaB inhibition of apoptosis in breast cancer cell lines. *Cancer Res* **67**, 6725-6736 (2007).
60. Verderio, C. et al. TI-VAMP/VAMP7 is the SNARE of secretory lysosomes contributing to ATP secretion from astrocytes. *Biol. Cell* **104**, 213-228 (2012).
61. Wu, B. et al. Up-regulation of Anxa2 gene promotes proliferation and invasion of breast cancer MCF-7 cells. *Cell Proliferation* **45**, 189-198 (2012).
62. Zhao, P. et al. Annexin II promotes invasion and migration of human hepatocellular carcinoma cells in vitro via its interaction with HAb18G/CD147. *Cancer Sci.* **101**, 387-395 (2010).
63. Koroseca, B., Glavac, D., Volavsekb, M. & Ravnik-Glavac, M. ATP2A3 gene is involved in cancer susceptibility. *Cancer Genet. Cytogenet.* **188**, 88 - 94 (2009).
64. Lei, J. et al. TC-1 Overexpression Promotes Cell Proliferation in Human Non-Small Cell Lung Cancer that Can Be Inhibited by PD173074. *PloS One* **9**, e100075 (2014).

65. Xu, H.-T. et al. TC-1 (C8orf4) expression is correlated with differentiation in ovarian carcinomas and might distinguish metastatic ovarian from metastatic colorectal carcinomas. *Virchows Archiv* **462**, 281 - 287 (2013).
66. Yang, Z.-Q., Moffa, A.B., Haddad, R., Streicher, K.L. & Ethier, S.P. Transforming properties of TC-1 in human breast cancer: Interaction with FGFR2 and β-catenin signaling pathways. *Int. J. Cancer* **121**, 1265-1273 (2007).
67. Kim, B. et al. TC1(C8orf4) Correlates with Wnt /β-Catenin Target Genes and Aggressive Biological Behavior in Gastric Cancer. *Clin. Cancer. Res.* **12**, 3541-3548 (2006).
68. Friedman, J.B., Brunschwig, E.B., Platzer, P., Wilson, K. & Markowitz, S.D. C8orf4 is a transforming growth factor B induced transcript downregulated in metastatic colon cancer. *Int. J. Cancer* **111**, 72-75 (2004).
69. Sunde, M. et al. TC-1 Is a Novel Tumorigenic and Natively Disordered Protein Associated with Thyroid Cancer. *Cancer Res.* **64**, 2766-2773 (2004).
70. Pibouin, L. et al. Cloning of the mRNA of overexpression in colon carcinoma-1: a sequence overexpressed in a subset of colon carcinomas. *Cancer Genet. Cytogenet.* **133**, 55-60 (2002).
71. Scanlan, M.J. et al. Antigens recognized by autologous antibody in patients with renal-cell carcinoma. *Int. J. Cancer* **83**, 456-464 (1999).
72. Cools, J. et al. Fusion of a Novel Gene, BTL, to ETV6 in Acute Myeloid Leukemias With a t(4;12)(q11 -q12;p13). *Blood* **94**, 1820-1824 (1999).
73. Nair, J. et al. Gene and miRNA expression changes in squamous cell carcinoma of larynx and hypopharynx. *Genes & Cancer* **6**, 328-340 (2015).
74. Chen, K. et al. Characterization of Tumor Suppressive Function of cornulin in Esophageal Squamous Cell Carcinoma. *PloS One* **8**, e68838 (2013).

75. Merkley, M.A. et al. 2D-DIGE proteomic characterization of head and neck squamous cell carcinoma. *Otolaryngology--head and neck surgery : official journal of American Academy of Otolaryngology-Head and Neck Surgery* **141**, 626-632 (2009).
76. Plebani, R. et al. Long-range Transcriptome Sequencing Reveals Cancer Cell Growth Regulatory Chimeric mRNA. *Neoplasia (New York, N.Y.)* **14**, 1087-1096 (2012).
77. Lv, Q. et al. DEDD Interacts with PI3KC3 to Activate Autophagy and Attenuate Epithelial ˈ Mesenchymal Transition in Human Breast Cancer. *Cancer Res.* **72**, 3238-3250 (2012).
78. Abe, S. et al. Copy number variation of the antimicrobial-gene, defensin beta 4, is associated with susceptibility to cervical cancer. *J Hum Genet* **58**, 250-253 (2013).
79. Semlali, A. et al. Expression and New Exon Mutations of the Human Beta Defensins and Their Association on Colon Cancer Development. *PloS One* **10**, e0126868 (2015).
80. Wygłędowska-Kania, M. et al. Defensin DEFB4A transcript level in the differentiation of keratoacanthoma, squamous and basal cell carcinomas. *Postępy Nauk Medycznych* **3**, 159 - 165 (2015).
81. Li, H. et al. DEPTOR has growth suppression activity against pancreatic cancer cells. *Oncotarget* **5**, 12811-12819 (2014).
82. Lai, E.-Y. et al. DEPTOR Expression Negatively Correlates with mTORC1 Activity and Tumor Progression in Colorectal Cancer. *Asian Pacific Journal of Cancer Prevention* **15**, 4589 - 4594 (2014).
83. Pei, L. et al. Overexpression of DEP domain containing mTOR-interacting protein correlates with poor prognosis in differentiated thyroid carcinoma. *Molecular Medicine Reports* **4**, 817 - 823 (2011).
84. Parvani, J.G. et al. Deptor Enhances Triple-Negative Breast Cancer Metastasis and Chemoresistance through Coupling to Survivin Expression()(). *Neoplasia (New York, N.Y.)* **17**, 317-328 (2015).

85. Peterson, T.R. et al. DEPTOR Is an mTOR Inhibitor Frequently Overexpressed in Multiple Myeloma Cells and Required for Their Survival. *Cell* **137**, 873-886 (2009).
86. Ying, H., Yu, Y. & Xu, Y. Cloning and Characterization of F-LANa, Upregulated in Human Liver Cancer. *Biochem. Biophys. Res. Commun.* **286**, 394-400 (2001).
87. Gu, H. et al. Association of endothelin-converting enzyme-1b C-338A polymorphism with gastric cancer risk: A case ' control study. *Eur. J. Cancer* **44**, 1253-1258 (2008).
88. Smollich, M. et al. On the role of endothelin-converting enzyme-1 (ECE -1) and neprilysin in human breast ancer. *Breast Cancer Research* **106**, 361 - 369 (2007).
89. Whyteside, A.R., Hinsley, E.E., Lambert, L.A., McDermott, P.J. & Turner, A.J. ECE-1 influences prostate cancer cell invasion via ET-1-mediated FAK phosphorylation and ET-1-independent mechanismsThis article is one of a selection of papers published in the two-part special issue entitled 20 Years of Endothelin Research. *Canadian Journal of Physiology and Pharmacology* **88**, 850-854 (2010).
90. Santos, C.R. & Schulze, A. Lipid metabolism in cancer. *FEBS J.* **279**, 2610 - 2623 (2012).
91. Walter, K. et al. DNA Methylation Profiling Defines Clinically Relevant Biological Subsets of Non ' Small Cell Lung Cancer. *Clin. Cancer. Res.* **18**, 2360 - 2373 (2012).
92. Kim, T.Y. et al. CRL4A-FBXW5 ' mediated degradation of DLC1 Rho GTPase-activating protein tumor suppressor promotes non-small cell lung cancer cell growth. *Proceedings of the National Academy of Sciences of the United States of America* **110**, 16868-16873 (2013).
93. NCBI, Vol. 2015 (2015).
94. Niemczyk, M. et al. Imprinted Chromatin around DIRAS3 Regulates Alternative Splicing of GNG12-AS1, a Long Noncoding RNA. *Am. J. Hum. Genet.* **93**, 224-235 (2013).

95. Nord, H. et al. Characterization of novel and complex genomic aberrations in glioblastoma using a 32K BAC array. *Neuro-Oncology* **11**, 803-818 (2009).
96. Baig, R.M. et al. Mutational Spectrum of Gelsolin and Its Down Regulation Is Associated with Breast Cancer. *Dis. Markers* **34**, 71-80 (2013).
97. Ni, X.-G. et al. The Ubiquitin-Proteasome Pathway Mediates Gelsolin Protein Downregulation in Pancreatic Cancer. *Molecular Medicine* **14**, 582-589 (2008).
98. K., H. he mechanism for reduced expression of gelsolin, tumor suppressor protein, in bladder ancer. *The Hokkido Journal of Medical Science* **78**, 29 - 37 (2 003).
99. LIU, J. et al. Concurrent Down-regulation of Egr-1 and Gelsolin in the Majority of Human Breast Cancer Cells. *Cancer Genomics - Proteomics* **4**, 377-385 (2007).
100. Hutti, J.E. et al. Phosphorylation of the tumor suppressor CYLD by the breast cancer oncogene IKKε promotes cell transformation. *Mol. Cell* **34**, 461-472 (2009).
101. Li, W. et al. IKBKE Upregulation is Positively Associated with Squamous Cell Carcinoma of the Lung In Vivo and Malignant Transformation of Human Bronchial Epithelial Cells In Vitro. *Medical Science Monitor : International Medical Journal of Experimental and Clinical Research* **21**, 1577-1586 (2015).
102. Péant, B. et al. IκB-Kinase-ε ︵IKKε/IKKi/IκBKε︶ expression and localization in prostate cancer tissues. *The Prostate* **71**, 1131-1138 (2011).
103. Hsu, S. et al. IKKε coordinates invasion and metastasis of ovarian cancer. *Cancer Res.* **72**, 5494-5504 (2012).
104. Lou, X. et al. MFAP3L activation promotes colorectal cancer cell invasion and metastasis. *Biochimica et Biophysica Acta (BBA) - Molecular Basis of Disease* **1842**, 1423-1432 (2014).
105. Lomakina, M.E. et al. Arpin downregulation in breast cancer is associated with poor prognosis. *Br J Cancer* **114**, 545-553 (2016).

106. Ishiyama, T. et al. OCIA domain containing 2 is highly expressed in adenocarcinoma mixed subtype with bronchioloalveolar carcinoma component and is associated with better prognosis. *Cancer Sci.* **98**, 50-57 (2007).
107. Nagata, C. et al. Increased expression of OCIA domain containing 2 during stepwise progression of ovarian mucinous tumor. *Pathology International* **62**, 471-476 (2012).
108. Chen, Q. et al. Poly r(C) Binding Protein -1 is Central to Maintenance of Cancer Stem Cells in Prostate Cancer Cells. *Cellular Physiology and Biochemistry* **35**, 1052-1061 (2015).
109. Zhang, H.-Y. & Dou, K.-F. PCBP1 is an important mediator of TGF-β-induced epithelial to mesenchymal transition in all bladder cancer cell line GBC-SD. *Mol. Biol. Rep.* **41**, 5519 - 5524 (2014).
110. Loilome, W. et al. PRKAR1A is overexpressed and represents a possible therapeutic target in human cholangiocarcinoma. *Int. J. Cancer* **129**, 34-44 (2011).
111. Checquolo, S. et al. Differential subcellular localization regulates c-Cbl E3 ligase activity upon Notch3 protein in T-cell leukemia. *Oncogene* **29**, 1463-1474 (20 10).
112. Kehlen, A. et al. Role of glutaminyl cyclases in thyroid carcinomas. *Endocrine-Related Cancer* **20**, 79-90 (2013).
113. Shields, D.J. et al. RBBP9: A tumor-associated serine hydrolase activity required for pancreatic neoplasia. *Proceedings of the National Academy of Sciences of the United States of America* **107**, 2189-2194 (2010).
114. Chen, M. & Geng, J.G. P-selectin mediates adhesion of leukocytes, platelets, and cancer cells in inflammation, thrombosis, and cancer growth and metastasis *Archivum Immunologiae et Therapiae Experimentalis* **54**, 75 - 84 (2006).
115. Cervelli, M. et al. Spermine oxidase (SMO) activity in breast tumor tissues and biochemical analysis of the anticancer spermine analogues BENSpm and CPENSpm. *BMC Cancer* **10**, 555-555 (2010).

116. Goodwin, A.C. et al. Increased Spermine Oxidase Expression in Human Prostate Cancer and Prostatic Intraepithelial Neoplasia Tissues. *The Prostate* **68**, 766-772 (2008).
117. Li, P., Lin, Y., Zhang, Y., Zhu, Z. & Huo, K. SSX2IP promotes metastasis and chemotherapeutic resistance of hepatocellular carcinoma. *J. Transl. Med.* **11**, 52-52 (2013).
118. Türeci, Ö. et al. The SSX-2 Gene, Which Is Involved in the t(X;18) Translocation of Synovial Sarcomas, Codes for the Human Tumor Antigen HOM-MEL-40. *Cancer Res.* **56**, 4766-4772 (1996).
119. Guinn, B.-a. et al. Humoral detection of leukaemia-associated antigens in presentation acute myeloid leukaemia. *Biochem. Biophys. Res. Commun.* **335**, 1293-1304 (2005).
120. Ren, D. et al. Phyllodes tumor of the breast: role of Axl and ST6GalNAcII in the development of mammary phyllodes tumors. *Tumor Biol.* **35**, 9603 - 9612 (2014).
121. Yoshida, N. et al. STX11 functions as a novel tumor suppressor gene in peripheral T-cell lymphomas. *Cancer Sci.* **106**, 1455-1462 (2015).
122. ZHOU, H. et al. Expression of TLR5 in Different Types of Non-small Cell Lung Cancer Cell Lines and its Activation Mechanism. *Chinese Journal of Lung Cancer* **18**, 8-15 (2015).
123. Kauppila, J.H., Mattila, A.E., Karttunen, T.J. & Salo, T. Toll-like receptor 5 (TLR5) expression is a novel predictive marker for recurrence and survival in squamous cell carcinoma of the tongue. *Br. J. Cancer* **108**, 638-643 (2013).
124. Pfirschke, C., Garris, C. & Pittet, Mikael J. Common TLR5 Mutations Control Cancer Progression. *Cancer Cell* **27**, 1-3.
125. Itkonen, H.M. et al. UAP1 is overexpressed in prostate cancer and is protective against inhibitors of N-linked glycosylation. *Oncogene* **34**, 3744-3750 (2015).
126. Kanojia, D. et al. Genomic landscape of liposarcoma. (2015).
127. Ito, E. et al. Uroporphyrinogen Decarboxylase Is a Radiosensitizing Target for Head and Neck Cancer. *Sci. Transl. Med.* **3**, 67ra67-67ra67 (2011).

128. Spybey, T. in Department of Physiology (University of Toronto, 2013).
129. Ye, Y. & Chen, S. Abstract 5000: Upregulation of WDR26 promotes breast cancer cell growth, migration and invasion via enhancing PI3K/AKT signaling. *Cancer Res.* **74**, 5000 (2014).
130. Ye, Y., Tang, X., Sun, Z. & Chen, S. Upregulated WDR26 serves as a scaffold to coordinate PI3K/ AKT pathway-driven breast cancer cell growth, migration, and invasion. (2016).
